# Solid-State Polymerization of Poly(Ethylene Furanoate) Biobased Polyester, III: Extended Study on Effect of Catalyst Type on Molecular Weight Increase

**DOI:** 10.3390/polym11030438

**Published:** 2019-03-06

**Authors:** Yosra Chebbi, Nejib Kasmi, Mustapha Majdoub, George Z. Papageorgiou, Dimitris S. Achilias, Dimitrios N. Bikiaris

**Affiliations:** 1 Laboratoire des Interfaces et Matériaux Avancés, Université de Monastir, Monastir 5000, Tunisia; yossrachebbi@gmail.com (Y.C.); mustaphamajdoub@gmail.com (M.M.); 2 Laboratory of Polymer Chemistry and Technology, Department of Chemistry, Aristotle University of Thessaloniki, GR-541 24 Thessaloniki, Greece; nejibkasmi@gmail.com (N.K.); axilias@chem.auth.gr (D.S.A.); 3 Chemistry Department, University of Ioannina, P.O. Box 1186, 45110 Ioannina, Greece

**Keywords:** poly(ethylene furanoate), solid-state polycondensation, catalysts, thermal properties, polyester

## Abstract

In this study, the synthesis of poly(ethylene furanoate) (PEF), catalyzed by five different catalysts—antimony acetate (III) (Sb Ac), zirconium (IV) isopropoxide isopropanal (Zr Is Ip), antimony (III) oxide (Sb Ox), zirconium (IV) 2,4-pentanedionate (Zr Pe) and germanium (IV) oxide (Ge Ox)—via an industrially common combination of melt polymerization and subsequent solid-state polymerization (SSP) is presented. In all reactions, proper amounts of 2,5-dimethylfuran-dicarboxylate (DMFD) and ethylene glycol (EG) in a molar ratio of DMFD/EG= 1/2 and 400 ppm of catalyst were used. Polyester samples were subjected to SSP procedure, under vacuum application, at different reaction times (1, 2, 3.5, and 5 h) and temperatures of 190, 200, and 205 °C. Carboxyl end-groups concentration (–COOH), intrinsic viscosity (IV), and thermal properties, via differential scanning calorimetry (DSC), were measured for all resultant polymers to study the effect of the used catalysts on the molecular weight increase of PEF during SSP process. As was expected, it was found that with increasing the SSP time and temperature, the intrinsic viscosity and the average molecular weight of PEF steadily increased. In contrast, the number of carboxyl end-groups content showed the opposite trend as intrinsic viscosity, that is, gradually decreasing during SSP time and temperature increase. It is worthy to note that thanks to the SSP process an obvious and continuous enhancement in the thermal properties of the prepared PEF samples was attained, in which their melting temperatures (*T*_m_) and degree of crystallinity (*X*_c_) increase progressively with increasing of reaction time and temperature. To predict the time evolution of polymers IV, as well as the hydroxyl and carboxyl content of PEF polyesters during the SSP, a simple kinetic model was developed. From both the theoretical simulation results and the experimental measurements, it was demonstrated that surely the Zr Is Ip catalyst shows the best catalytic characteristics compared to all other used catalysts herein, that is, leading in reducing—in a spectacular way—the activation energy of the involved both transesterification and esterification reactions during SSP.

## 1. Introduction

In recent years, the interest to develop biobased ecofriendly polymeric materials derived from biomass—instead of petroleum-based polymers—has emerged with renewed strength. The latter was stimulated by the global depletion of fossil fuel resources and fluctuations in oil prices, as well as serious environmental pollution [1,2,3,4,5,6,7]. Moreover, the USA and European legislative landscapes are changing in favor of renewable resource-based products against fossil-based ones [8,9]. In this context, and thanks to the rapid development of the chemical industry and the optimization of biorefining processes [10,11], large-scale production of renewable resource-derived monomers has been developed in the last few years and are now widely used as starting building blocks for biobased polymers production [12,13,14]. Among them, 2,5-furandicarboxylic acid (FDCA), which is widely recognized as the promising biobased substitute to terephthalic acid (TPA) [15], and its large-scale production have been realized recently. Thanks to its special chemical structure, FDCA has been selected as one of the 12 top value-added chemicals by the US Department of Energy [16]. In fact, for the above-mentioned reasons, a paradigm shift has emerged towards synthesizing of different homopolymers derived from the renewable-based aromatic diacid monomer (FDCA) and several diols [17,18,19,20,21,22,23,24,25,26,27]. In this sense, FDCA is on the way of industrialization and commercialization by many international companies, including Corbion, Avantium, ADM, BASF, and DuPont [28]. This is due to its great potential in high-performance renewable polymers synthesis; thereby, they can be served as alternatives to their current commercial fossil-based analogs. As a relevant example of sustainable FDCA-based polymers from renewable resources, poly(ethylene furan dicarboxylate) (PEF), produced from either 2,5-furandicarboxylic acid (2,5-FDCA) or its dimethyl ester (DMFD) and ethylene glycol, is the most promising biobased polyester. The latter is a 100% renewable-based alternative to its commercial petrochemical counterpart, polyethylene terephthalate (PET) [15]. Extensive research has escalated towards PEF since the last decade, and its most relevant achievements and its historical progress have been intensively described in two recent extended reviews [29,30]. The most spotlighted member of furanoate polyesters family—PEF—is of significant and increased interest, owing to its pioneering features and its entirely renewable source. This is clearly evident from the exhaustive and intensive studies, which have been conducted on this commercial polyester. Notably, outstanding properties of PEF polyester were revealed, such as greatly improved thermal stability up to 320 °C [31,32], a superior barrier performance, where an impressive 19-fold and 10–27-fold reduction has been reported in CO_2_ and O_2_ permeability, respectively, for PEF with respect to its oil-based counterpart PET [33]. Other appealing characteristics, including better greenhouse gas balance and reduced carbon footprint [34], excellent mechanical properties [35], in addition of an ability to formulate in fibers, films, and most bottles make PEF an appropriate commodity for ecopackaging applications, and thereby it can serve as a promising substituent for PET in the near future [36]. Recently, the techno-economic feasibility of biorefinery to adopt biobased PEF polyester production has been definitely proven. In this context, as a result of all its unique aforementioned features, manufacturing of PEF has been begun in 2010 by Avantium in Netherlands for typical applications such as fibers, films, and in particular for packaging of alcoholic beverages, water, and soft drinks, among others, using the Avantium’s YXY technology [37]. This is considered a key point in favor of this promising polyester, that is, it has now enabled prospects for its industrial-scale production, hence creating its own market.

Although much work has recently appeared on PEF by highlighting on its better features compared with its analog, PET, this promising polyester suffers from some drawbacks, which renders its industrialization process uneconomical. Particularly, production at high molecular weight PEF, which ensures the safe processing of the resulting polyester without any deterioration of its mechanical properties, besides overcoming of its high brittleness [38] and undesired yellow discoloration [39], is a challenge for both academic and industrial communities. The catalyst type used, which has an important role in molecular weight increase, as well as the decomposition of 2,5-FDCA at high reaction temperature during melt polycondensation reactions, are deemed to become one of the main causes to produce PEF with low molecular weight.

Little research has been devoted to PEF synthesis, which involves studying the effect of different experimental conditions during the two-stage melt polycondensation process on its molecular weight increase. In this respect, Gruter et al. [40] claimed the synthesis of PEF via melt polycondensation using two catalysts: titanium (IV) Isopropoxide and a butyltin (IV) tris(octoate)-tris(nonylphenyl)phosphite mixed catalyst system. This systematic study was carried out on small scale in polycondensation films reactors. The authors reported that no increase in molecular weight was detected using a concentration of the first catalyst over 0.4 µmol, while a higher concentration of the second catalyst system leads to higher *M*_n_. Moreover, results revealed that the addition of tris(nonylphenyl)phosphite may serve as a heat stabilizer, to reduce, significantly, the coloration of the obtained polyester, PEF. This assessment has mainly tackled the discoloration of PEF polyester by varying the used catalyst type.

To get high molecular weight polyesters with enhanced properties suitable for several applications (e.g., films, bottles, and fiber production), a well-known technique and extensively exploited in the industry as a third-step to polyesters, which is solid-state polymerization (SSP) [41]. This method is carried out by heating the partially crystalline polymer at a temperature between its glass transition temperature (*T*_g_) and its melting point (*T*_m_). The latter is greatly used for PET manufacturing to overcome its low molecular weight [42,43,44,45,46,47,48,49]. This postcondensation technique is ideally attractive and environmentally sound process compared to conventional melt polycondensation, as it is a solvent-free method and no toxic wastes are released upon its execution. Furthermore, as one of its most outstanding features, SSP requires uncomplicated and inexpensive equipment, as well as it can be implemented under relatively mild conditions, thereby fewer side reactions and thermal degradation can occur.

PEF is intriguing to both the scientific and industrial communities and it has drawn a keen interest as biobased polymer in food and beverage packaging. Much work has been dedicated to PEF investigation, showing thus the major advances and breakthroughs in this topic. In this context, significant number of studies addressed on its barrier [36,50,51,52], thermal [31,33,53,54,55], and mechanical [35,56] properties, as well as investigations have dealt with the biaxial orientation [57], kinetics and dynamics of crystallization [58,59,60,61], synthesis, and structural characterization of this promising polyester [24,54,62,63,64,65,66]. 

Apart from several studies recently published which expanded PEF polyester’s properties panorama, only a few investigations exclusively dealt with the industrially relevant technique (SSP) of PEF, despite its benefits with respect to the manufacturing of high molecular weight polyester. To date, as far as we know, only three earlier appraisals [15,67,68], along with our previous studies [69,70,71], recently published, have been intended to investigate SSP of PEF as a subsequent third stage after two-step melt polycondensation procedure. In this regard, Knoop et al. [56] point out that the high molecular weight of PEF polyester was successfully achieved by applying SSP procedure under reduced pressure, using Ti(IV)-isopropoxide as catalyst. In this study, the synthetic challenge of high PEF polyester degree of polymerization was accomplished during several hours, in which the *M*_n_ of the obtained polymer was gradually increased from 25,000 g/mol to 83,000 g/mol after 24 and 72 h SSP, respectively, at 180 °C. In fact, the crystallization investigation, as well as its influence on the mechanical properties of high molecular weight polyester PEF, has been evaluated thoroughly by the authors of this contribution. In the same vein, as reported in Hong’s work [67], PEF has been subjected to SSP, and results exhibited an increase in IV values of the polyester sample from 0.6 to 0.64 and 0.72 dL/g after 24 and 48 h of SSP reaction time, respectively.

As one of very limited studies reporting the effect of the catalyst type on molecular weight increase of poly(ethylene furanoate) using solid-state polymerization, the aim of this work was to study the feasibility of PEF SSP, using a series of catalysts—Antimony Acetate (III) (Sb Ac), Zirconium (IV) Isopropoxide Isopropanal (Zr Is Ip), Antimony (III) Oxide (Sb Ox), Zirconium (IV) 2,4-pentanedionate (Zr Pe), and Germanium (IV) Oxide (Ge Ox). The effect of the catalyst type, along with the influence of the temperature and reaction time on the molecular weight increase of the prepared polyester PEF, was investigated in detail using both experimental measurements and a simple kinetic theoretical model. Notwithstanding the increasing number of publications towards PEF study, to the best of our knowledge, such work, which has not yet been reported, is essential nowadays to enable large-scale industrial production and commercial applications of PEF.

## 2. Experimental

### 2.1. Materials

2,5-furan dicarboxylic acid (2,5-FDCA, purum 97%), ethylene glycol (99.8%), Antimony acetate (III) (Sb Ac, 99.99%), Antimony (III) Oxide (Sb Ox, >99%), Germanium (IV) oxide (Ge Ox, >99.99), Zirconium (IV) 2,4-Pentanedionate (Zr Pe, 97%), and Zirconium(IV) Isopropoxide Isopropanal (Zr Is Ip, 99.9%) catalysts were purchased from Aldrich Co. (Chemie GmbH, Unna, Germany). All other solvents and materials used were of analytical grade. 

### 2.2. Synthesis of 2,5-Dimethylfuran-dicarboxylate(DMFD)

Dimethyl 2,5-dimethylfurandicarboxylate (DMFD) was prepared following the reported procedure [55]. Briefly, in a random flask (500 mL), a mixture of 15.6 g of 2,5-furandicarboxylic acid, 2 mL of concentrated sulfuric acid and 200 mL of anhydrous methanol and was refluxed for 5 hours. The excess of the methanol was distilled and then the solution was filtered via a disposable Teflon membrane filter (Chemie GmbH, Unna, Germany). Dimethyl ester was precipitated during filtration as white powder. After cooling, 100 mL of distilled water was added to the powder. During stirring, a solution of Na_2_CO_3_ 5% *w*/*v* was added to neutralize partially the dispersion while pH was measured continuously. The white powder was collected by filtration and the solid was washed several times with distilled water and finally dried. The isolated dimethyl ester was recrystallized using a mixture of 50/50 *v*/*v* methanol/water, and white needles of DMFD were isolated in 83% yield. The melting point of the prepared DMFD herein is 109.6 °C. This is in good agreement with what was reported in a previous study of Knoop et al. [56] (*T*_m_: 109.76 °C).

### 2.3. Polyester Synthesis

PEF samples were synthesized through the two-step melt polycondensation (esterification and polycondensation) in a glass batch reactor following the same procedure reported in our previous work [72]. Briefly, predetermined amount of DMFD and ethylene glycol in a molar ratio of diester/diol = 1/2 were charged with 400 ppm of each catalyst relative to the amount of DMFD (Sb Ac, Sb Ox, Ge Ox, Zr Pe, and Zr Is Ip) into the reaction tube of the polyesterification apparatus. In the first stage, the reaction mixture was heated under controlled N_2_ atmosphere for 2h at 160 °C, at 170 °C for an additional 1h, and finally it was left to proceed for 1h at 180–190 °C. This first stage (transesterification) was considered complete after the collection of almost all the theoretical amount of CH_3_OH, which was removed by distillation from the reaction mixture in a graduated cylinder. In the second polycondensation step, a high pressure of 5.0 Pa was gradually applied over a period of 30 min to remove the excess EG diol, to avoid excessive foaming and, moreover, to minimize oligomer sublimation, which presents a problem during the melt polycondensation. The reaction mixture was gradually raised to 230 °C, while stirring speed was set at 720 rpm. The reaction was maintained at this temperature for 2 h. After the polycondensation reaction was finished, PEF sample was easily removed from the reactor, milled, and washed with methanol.

### 2.4. Solid-State Polycondensation

Solid-state polymerization (SSP) was performed using a reaction apparatus involving five volumetric flasks (100 mL), which were connected to a vacuum line, and were immersed in a sodium nitrite/potassium nitrate thermostated bath, having a precision within ±0.5 C. 2 g of crystallized PEF with a particle size of −0.40 + 0.16 mm were introduced in each one of the volumetric flasks under vacuum, stabilized beneath 3 and 4 Pa. The reaction temperature was maintained constant at 190, 200 or 205 °C. The flasks were withdrawn from the bath after 1, 2, 3.5, and 5h for analysis of the PEF samples’ intrinsic viscosity (IV), to identify the molecular weight of the PEF polyester, as well as measuring the carboxyl end-group concentration (COOH).

### 2.5. Polyester Characterization

#### 2.5.1. Intrinsic Viscosity Measurement

For intrinsic viscosity [η] measurements, a mixture of phenol/tetrachloroethane (60:40 *w/w*) was used to dissolve PEF samples (1 wt %) at 90°C. To achieve a complete dissolution, the latter were maintained under stirring in the above mixture of solvents at this temperature for 10 min. After cooling to room temperature and filtration using a disposable membrane filter (Teflon) to eliminate any nonsoluble materials, their flow time was measured at 25 °C using an Ubbelohde viscometer (Schott Gerate GMBH, Hofheim, Germany). The intrinsic viscosity [η] of each PEF sample was calculated using the following Solomon–Ciuta equation.

[η] = [2{*t/t_0_* − ln(*t/t_0_*) − 1}] ^1/2^/*c* (1)

where *t* is the flow time of solution, *t_0_* is the flow of pure solvent, and *c* is the concentration of the solution. For each sample, the average value was determined after performing three different measurements.

#### 2.5.2. Molecular weight

The number average molecular weight (${\bar{M}}_{n}$) of the PEF samples was determined from the measured [η] values, using the Berkowitz equation [73], as was modified in our previous report [74]:

(2) $${\bar{M}}_{n}=3.29\times 10^{4}{\left[\eta \right]}^{1.54}$$

#### 2.5.3. End-Group Analysis

Carboxyl end-group content (C.C.) of the PEF samples was determined according to Pohl’s method by titrating a solution of the PEF in chloroform/alcohol mixture. A standard solution of NaOH in benzyl alcohol and phenol red as indicator was used [75]. For each sample, three different measurements have been done and the average C.C. value was calculated.

#### 2.5.4. Wide Angle X-Ray Diffraction Patterns (WAXD)

X-ray diffraction measurements of the prepared PEF polyester samples with different catalysts before SSP, in the form of powder, were performed in the wide-angle region in the angle (2*θ*) range from 5° to 60°, at steps of 0.05°, and counting time of 5 s/step, using a MiniFlex II XRD system from Rigaku Co. (Tokyo, Japan) with CuKα radiation (λ = 0.154 nm). 

#### 2.5.5. Differential Scanning Calorimetry (DSC)

Thermal analysis of PEF polyesters was carried out using a Perkin-Elmer, Pyris Diamond DSC differential scanning calorimeter (Perkin-Elmer, Waltham, MA, USA), calibrated with high purity metal standards (indium and zinc). For each measurement, a PEF sample of 6 ± 0.1 mg was placed in aluminum pan and was then heated from 30 to 250 °C at a scanning rate of 20 °C.min^-1^ under N_2_ flow (50 mL/min). The melting temperature (*T*_m_), the glass transition temperature (*T*_g_), and the heat fusion (DHm) of the PEF polyesters were measured from these scans.

## 3. Modeling the PEF SSP Kinetics

### 3.1. Reaction Mechanism

The reactions occurring during SSP of PEF polyester include polycondensation/transesterification, esterification, thermal degradation, and side reactions of vinyl end-groups [43]. The latter are illustrated in the following Equations (3)–(6), in which *k_1_*, *K_1_* and *k_2_*, *K_2_* indicate the forward and equilibrium rate constants of transesterification and esterification reactions, respectively, while *k*_v_ and *k*_d_ refer to the kinetic rate constants of the polycondensation of vinyl end-groups and degradation reactions, which are considered one way. It should be pointed that the reverse kinetic rate constants *k*_1_’ and *k*_2_’ in Equations (3) and (4) have been replaced by their equivalent *k*_1_’ = *k*_1_/*K*_1_ and *k*_2_’ = *k*_2_/*K*_2_ from the definition of the equilibrium rate constants as, *K*_1_ = *k*_1_/*k*_1_’ and *K*_2_ = *k*_2_/*k*_2_’, respectively.

Polycondensation/transesterification

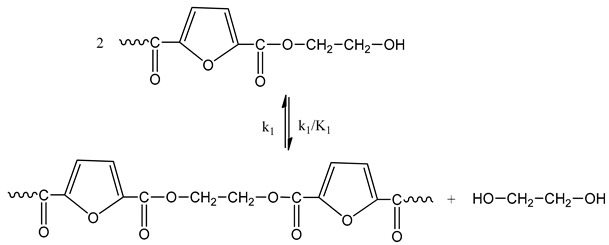
(3)

Esterification

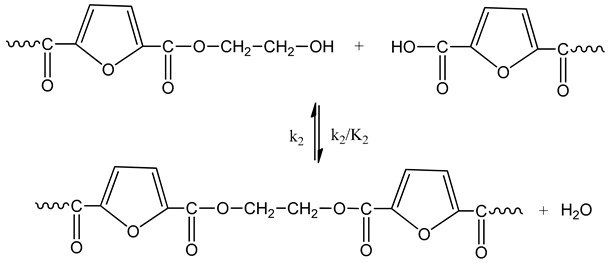
(4)

Thermal degradation

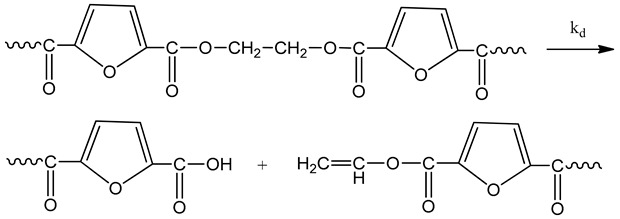
(5)

Polycondensation of vinyl end-groups

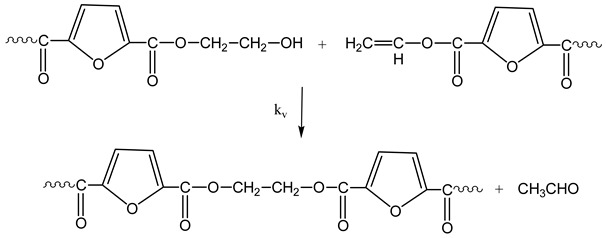
(6)

The molecular weight of the PEF polyester is increased by two reactions: the first one is polycondensation/transesterification (Equation (3)), where two hydroxyl end-groups react, and ethylene glycol was produced. The second reaction is the esterification (Equation (4)), in which a carboxyl end-group reacts with a hydroxyl, and H_2_O is released as by-product. In contrast, when thermal degradation occurs (Equation (5)), the molecular weight of the polymer can be decreased by an ester bond cleavage in the macromolecular chain, thereby generating a carboxyl end-group and a vinyl ester end-group. In addition, the vinyl ester end-group may take part in side reactions with a hydroxyl end-group, resulting in the molecular weight increase (Equation (6)). The overall reaction rate is influenced by a combination of intrinsic reaction kinetics, diffusional limitations of the reactive end-groups, change of polymer degree of crystallization, and of the desorbing volatile byproducts (i.e., water and glycol) [76].

### 3.2. Simplified Mathematical Model

The problem of modeling the SSP kinetics is quite complicated, since, besides chemical kinetics, describing the rate of change of the species’ concentration as a function of time, diffusion phenomena should be included, which results in additional variation with the distance from the interface [43]. Thus, two independent variables are presented, resulting in a set of partial differential equations that must be solved, including several diffusional, kinetic, and crystallization parameters [77]. Adopting such complicated models to simulate a few experimental data points is out of any physical meaning. Since in this investigation, only five data points were measured at each experimental condition, a simple kinetic modeling approach was adopted after Agarwal et al. [78,79]. This approach was initially developed for the SSP of PET, and successfully applied by our research group in modeling the SSP of PEF with nanoadditives, as well as of PET with several nanoadditives [69,80,81,82].

The following assumptions were made in order to develop the mathematical model.

- All kinetic rate constants are regarded independent of polymer chain length (only end-group reactivity is considered).
- The backward reactions in Equations (3) and (4) are eliminated. This is due to the very fast removal of the ethylene glycol and water produced in the reaction mixture, caused by the high vacuum application (beneath 3 and 4 Pa).
- Since polycondensation occurs at relatively low temperatures (i.e., 190–205 °C), no side reactions for the formation of acetaldehyde or thermal degradation are considered (Equations (5) and (6) are excluded).
- Diffusional limitations on account of desorbing volatile species are ignored.
- Then, the rate of change of hydroxyl ([OH]) and carboxyl ([COOH]) end-groups can be described by the following expressions [78,79].

(7) $$\frac{d{\left[\mathrm{OH}\right]}_{t}}{dt}=-2k_{1}{\left[\mathrm{OH}\right]}_{t}^{2}-k_{2}{\left[\mathrm{COOH}\right]}_{t}{\left[\mathrm{OH}\right]}_{t}$$

(8) $$\frac{d{\left[\mathrm{COOH}\right]}_{t}}{dt}=-k_{2}{\left[\mathrm{COOH}\right]}_{t}{\left[\mathrm{OH}\right]}_{t}$$

where [COOH]*_t_* and [OH]*_t_* denote the actual ‘true’ carboxyl and hydroxyl end-group concentration, respectively.

The term “actual hydroxyl and carboxyl end-groups” was introduced by Agarwal and coworkers [78,79], in order to account for the rapid slowdown in SSP kinetics at high [η] values. Accordingly, a part of the hydroxyl ([OH]) and carboxyl end-groups ([COOH]) were considered to be rendered temporarily inactive (denoted as [OH]*_i_* and [COOH]*_i_*) and the actual concentration of OH and COOH in Equations (7) and (8) can be expressed as

[OH]*_t_* = [OH] − [OH]*_i_* (9)

[COOH]*_t_* = [COOH] − [COOH]*_i_* (10)

where [COOH], [OH] and [COOH]*_i_,* [OH]*_i_* denote the concentration of the total and temporarily inactivated COOH and OH end-groups, respectively.

Furthermore, the number average molecular weight can be expressed as

(11) $${\bar{M}}_{n}=\frac{2}{\left[\mathrm{COOH}]+[\mathrm{OH}\right]}$$

Then, Equations (7) and (8), together with Equations (2) and (9)–(11), constitute a set of ordinary differential equations which can be readily solved numerically using varying step-size Runge–Kutta method, to give results on the variation of the intrinsic viscosity and the concentration of carboxyl and hydroxyl end-groups as a function of time. Four adjustable parameters—[OH]*_i_*, [COOH]*_i_*, *k*_1_, and *k*_2_—are estimated at each experimental condition by simultaneous fitting of the values of all the three variables (IV, [OH], and [COOH]) to the experimental data points as a function of time.

## 4. Results and Discussion

**Figure 1:**
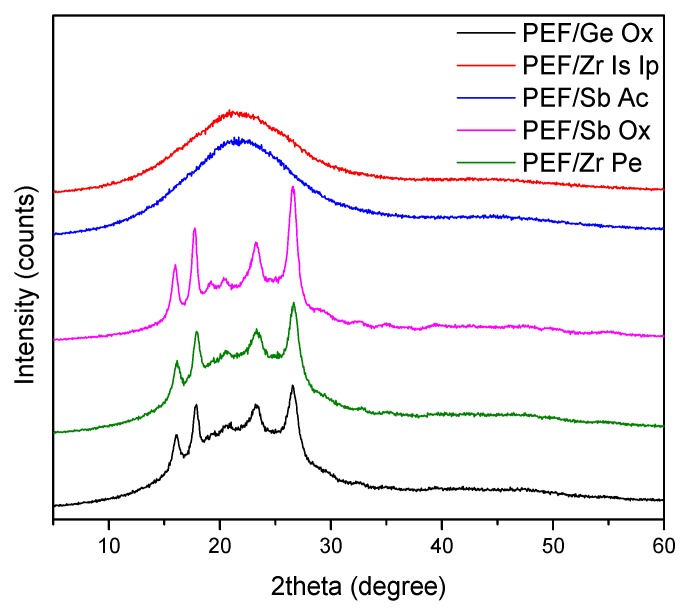
X-ray diffraction analysis (WAXD) patterns of as received poly(ethylene furanoate) (PEF) samples after melt polycondensation using five different catalysts (Ge Ox, Zr Is Ip, Sb Ac, Sb Ox, and Zr Pe).

In this work, emphasis is given to study the effect of five different catalysts, namely Ge Ox, Zr Is Ip, Sb Ac, Sb Ox, and Zr Pe, on solid-state polymerization (SSP) of PEF. The latter were first synthesized by melt polycondensation according to our previous work [83] and their intrinsic viscosities oscillate between 0.25 and 0.40 dL/g. X-ray diffraction analysis (WAXD) was conducted to assess the crystalline structure of the resulting PEF materials after melt polycondensation. As can be seen from Figure 1, only PEF polyesters prepared with (Sb Ac) and (Zr Is Ip) catalysts are completely amorphous, while the diffractogram of the other three samples proves their semicrystalline nature, all exhibiting very similar diffraction peaks at 16.1°, 17.7°, 19.1°, 20.4°, 23.3°, and 26.6°, as stated in our earlier reports [33,84]. This finding confirms that the crystallinity of PEF depends on the used catalyst type. After melt polycondensation, SSP was carried out under vacuum at different temperatures (190, 200, and 205 °C) for 1, 2, 3.5, and 5 h. Polyesters samples for SSP should be crystallized at 170 °C for 6 h before SSP. This step, used always in PET before SSP, is mandatory to avoid any PEF amorphous regions that stuck each other. 

### 4.1. Kinetic Study of the Solid-State Polymerization of PEF

SSP is an important process widely applied after melt polymerization to improve the mechanical and rheological properties of polymers. Therefore, it is also a common approach to overcome its low molecular weight. The current work is a continuation of a study of the temperature and time evolution during SSP on polymer’s hydroxyl and carboxyl content as well as the intrinsic viscosity. 

**Figure 2:**
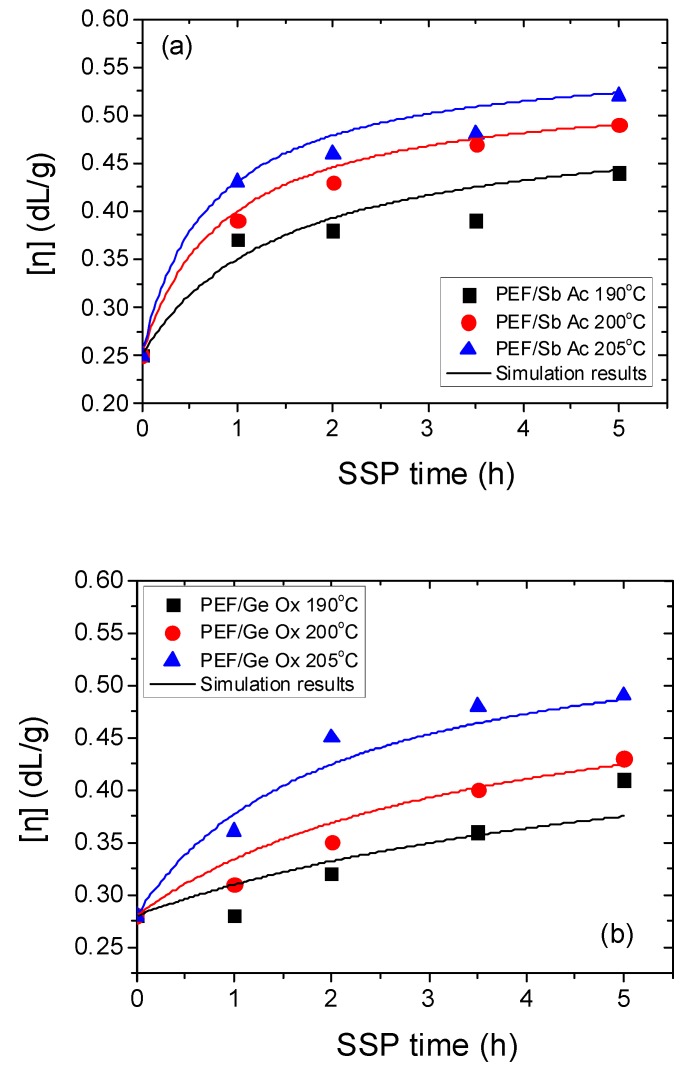

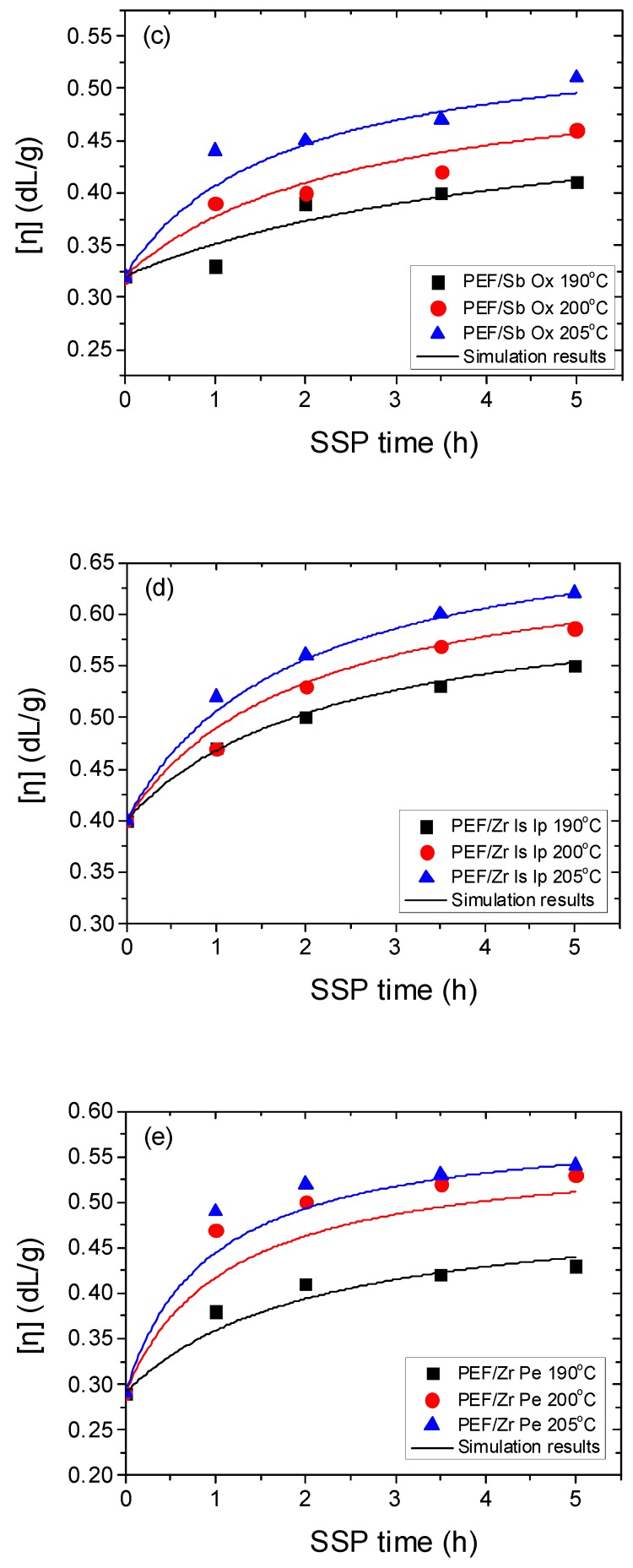
Variation of the intrinsic viscosity with time during the solid-state polymerization (SSP) of PEF using different catalysts—Sb Ac (**a**), Ge Ox (**b**), Sb Ox (**c**), Zr Is Ip (**d**), and Zr Pe (**e**)—at different temperatures. Continuous lines represent the theoretical kinetic model simulation results.

SSP was carried out at temperatures 190, 200 and 205°C for 1, 2, 3.5, and 5 h. Figure 2 shows the effect of time and temperature during solid-state polymerization on the variation of the intrinsic viscosity with time for all the polyesters samples investigated. As it can be seen, regardless of the type of catalyst used, with increasing time or temperature, the intrinsic viscosity significantly increases, for example, for PEF/Zr Pe, the intrinsic viscosity starts from 0.38 dL/g to achieve after 5 h of polycondensation 0.43 dL/g at 190°C or 0.54 dL/g at 205°C. Using Ge Ox as catalyst, the intrinsic viscosity increased from 0.28 dL/g, to 0.41, 0.43, and 0.49 dL/g after 5 h of solid-state polycondensation respectively at 190, 200, and 205°C. The use of this catalyst during SSP results in a polymer having the lowest intrinsic viscosity; whereas, the IV of PEF when using Zr Is Ip was the highest. In the latter case, IV starts from 0.47 dL/g at 190°C to reach 0.60 dL/g after 5 h at 205 °C. Using Sb Ox, at 190°C the IV was 0.33 dL/g, and increased progressively with temperature during time to achieve after 5h at 205°C the value of 0.51 dL/g. For PEF/Sb Ac, an increasing intrinsic viscosity was observed, starting from 0.37 dL/g to reach 0.52 dL/g after 5h at 205°C. As it was expected, during SSP, which is performed in the crystalline state of the polyesters, the concentration of the polymer chain and the mobility of the end-group was consequently enhanced, this is certainly due to increasing of the end-groups reactivity and the concomitant diffusion of diol which leads to chain extension. However, the study of intrinsic viscosity values affords a general idea about the activity of each catalyst; thus, at 190°C, the evolution during time of the intrinsic viscosities was very leisurely despite the type of catalyst. This is was expected because the esterification and transesterification reactions were very low at this temperature, which is 20–30 °C below the melting point of PEF. Overall, those reactions are controlled by the diffusion of two products (water and ethylene glycol), with the latter became tardy at low SSP temperature. For the same reasons, an increasing of the IV values was distinguished at 200 °C. However, at 205°C, which is closer to the melting point of PEF, whatever the catalyst used, we noticed during the first two hours a rapid increase of the IV. As a consequence of this high temperature, it becomes more straightforward for the hydroxyl and carboxyl end-groups to react together because of the higher mobility of the macromolecular chains of PEF; which consequently enhances the molecular weight of the polyester. Based on what has been discussed above about the IV variation, we can conclude that the temperature is one of the most important parameters which could affect the molecular weight of the polymer. The number average molecular weight (*M*_n_) of all synthesized PEF and the corresponding number average of the degree of polymerization are presented in Table 1. These values were estimated from the experimental data of IV using Equation (2). As it was anticipated, *M*_n_, such as IV, depends on time and temperature. It is noteworthy that all the values may not be precise since in literature Equation (2) is available for PET. However, from this data, it seems that using Zr Is Ip leads to a PEF polyester with higher molecular weight than the other catalysts used previously even higher than PEF with DBTO, TBT and TIS as it was found in our previous work [70]. In contrast, Ge Ox catalyst led to the lowest *M*_n_ values.

**Figure 3:**
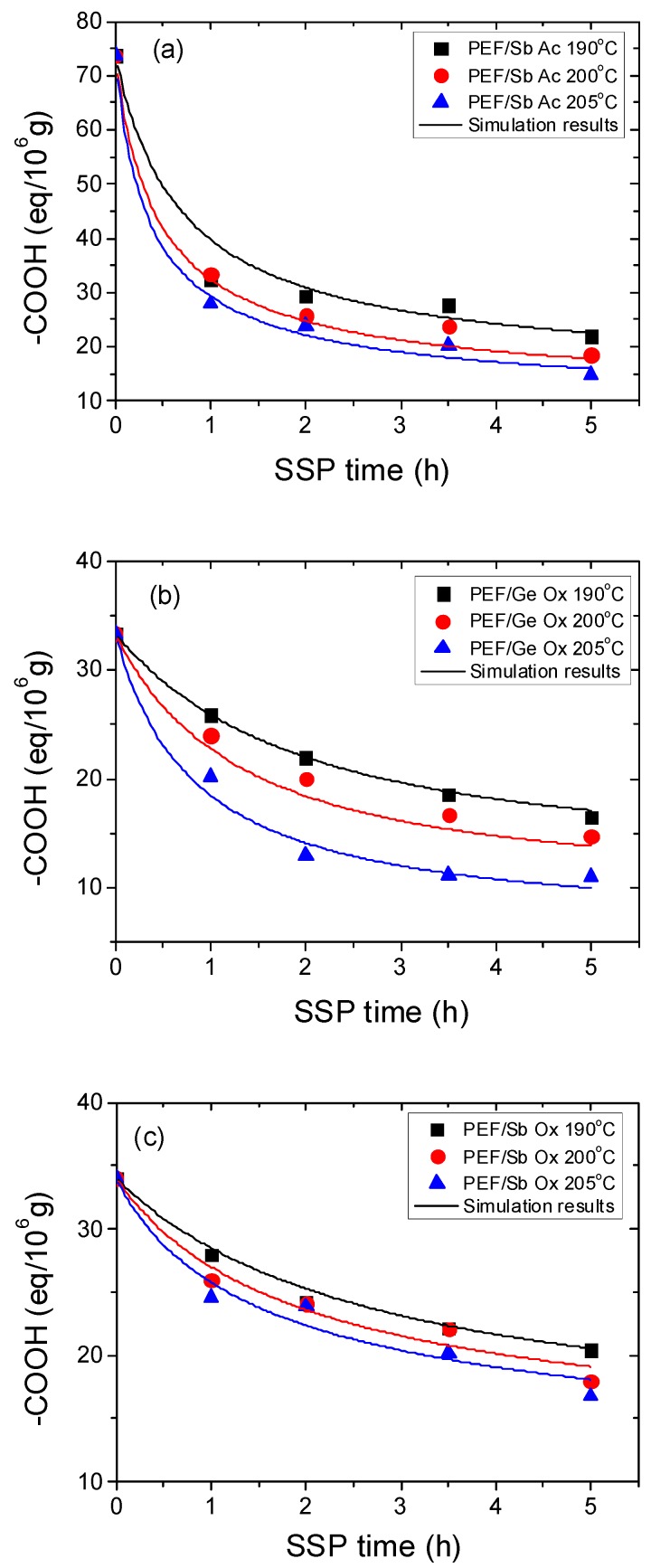

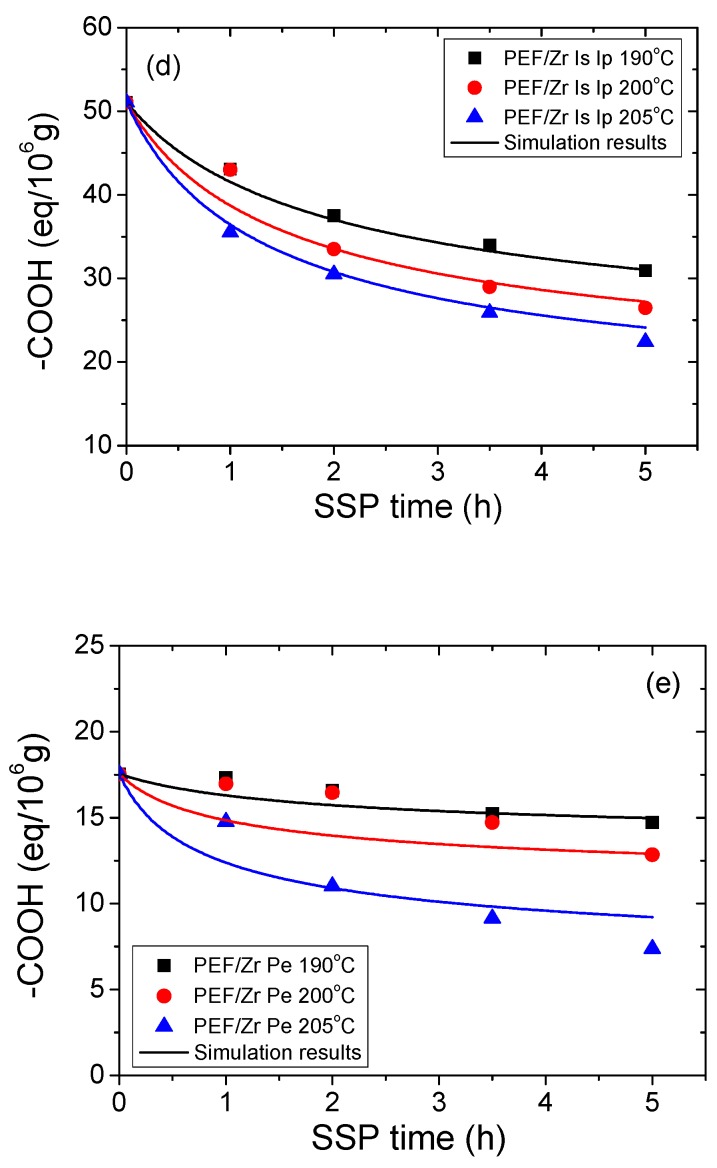
(–COOH) Variation with time during SSP: PEF/Sb Ac (**a**), PEF/Ge Ox (**b**), PEF/Sb Ox (**c**), PEF/Zr Is Ip (**d**), and PEF/Zr Pe (**e**) at different temperatures. Continuous lines represent the theoretical kinetic model simulation results.

The second parameter used to illuminate the effect of SSP time and temperature is the carboxyl end-group analysis (–COOH) which is performed on all PEF samples. Figure 3 displays the variation of the end-group concentration during time when the catalyst and the temperature are modified. It is noteworthy that, regardless the type of catalyst used, all PEF polyesters have a small amount of carboxyl end-groups. According to the literature, the determined carboxyl end-groups for neat PEF was (36 eq/10^6^ g). As it can be seen from the experimental curves, at temperatures close to the melting point of PEF, the trend is even more obvious, it shows the fastest decrease no matter which catalyst we use. However, for PEF using Ge Ox, Sb Ac, and Sb Ox the carboxyl end-groups starts approximately at 205 °C from 25 to reach 15 eq/10^6^g after 5 hours. Using Zr Is Ip as a catalyst, which provides the highest molecular weight of PEF, the carboxyl end-groups at 205°C begin at 51 to attain, after 5 h of SSP, 22 eq/10^6^g, whereas the concentration of the carboxyl end-groups for PEF with Zr Pe was reduced to 7.38 eq/10^6^g at 205°C after 5 hours. After a general inspection about the evolution of the carboxyl end-groups values, it is notable that the latter depend essentially on time, thus with increasing time it was continuously reduced, whereas with increasing temperature the variation was less important. As it can be seen from Figure 3, this reduction was more important at 205 °C wherein the deviation angle of curves during time was more important, whereas at 200 and 190 °C it was less sharp and almost linear. All PEF polyesters were performed via the two-step melt polycondensation thus the bis-hydroxyethylene furanoate formed at the first stage was conducted to a second stage of polycondensation in order to prepare poly(ethylene furanoate) which contain as end-groups only hydroxyl. The existence of the carboxyl end-groups can be elucidated by the existence of decomposition reactions that are taken place during melt polycondensation which lead to carboxyl and vinyl end-groups (Equation (5)). According to Wang et al. [45], another amount of carboxyl end-groups could be acquired because of the reaction of esterification taking place (Equation (4)); therefore, the molecular weight of PET also was enhanced while the carboxyl end-groups was decreased because of the reaction of esterification taking place.

**Figure 4:**
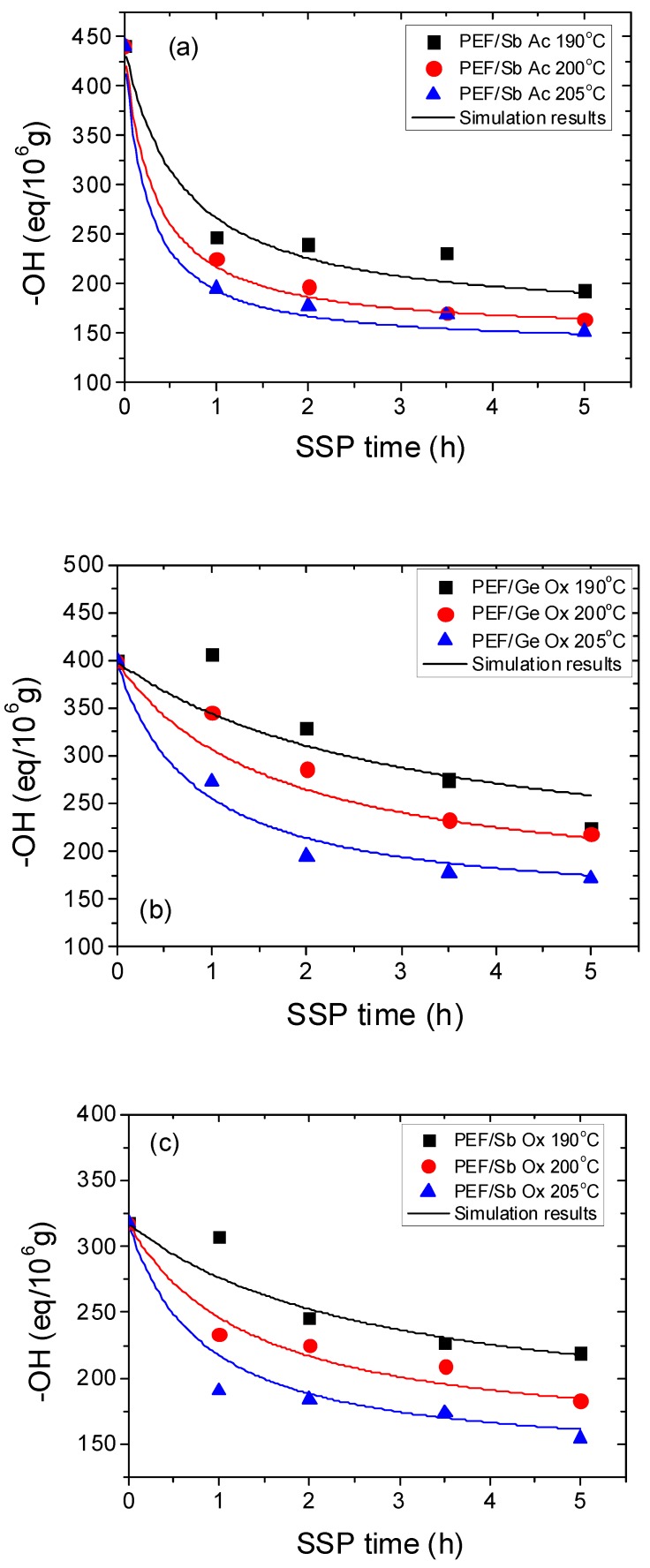

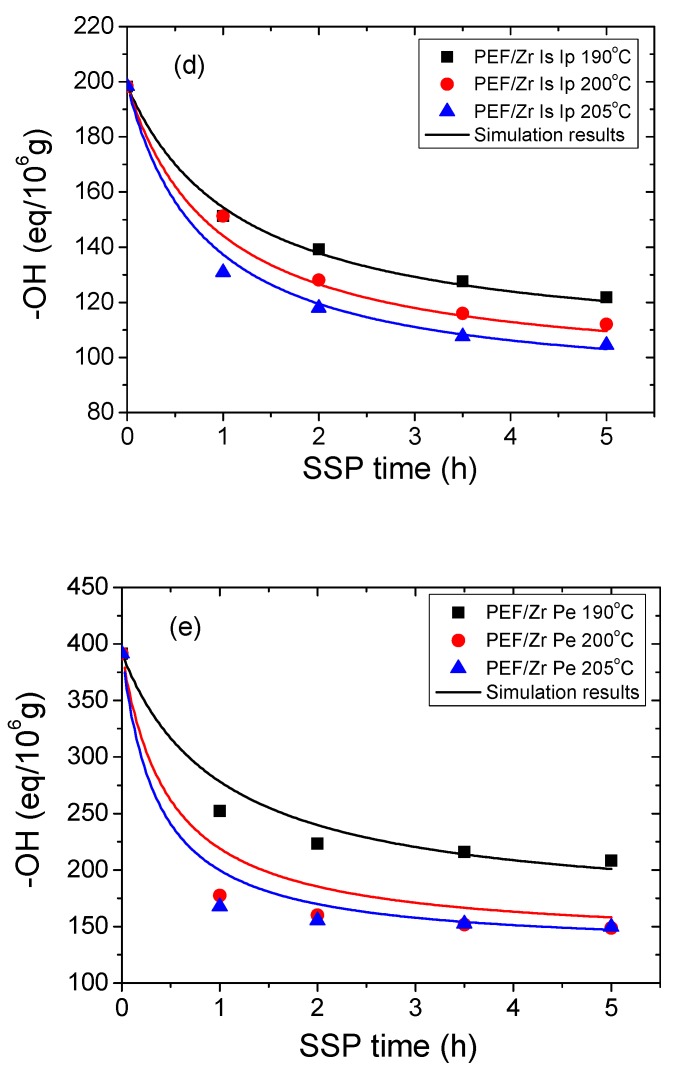
Variation of hydroxyl end-groups with time during PEF/Sb Ac (**a**), PEF/Ge Ox (**b**), PEF/Sb Ox (**c**)**,** PEF/Zr Is Ip (**d**)**,** and PEF/Zr Pe (**e**) SSP at different temperatures. Continuous lines represent the theoretical kinetic model simulation result.

Moreover, hydroxyl end-groups content (–OH) for all PEF polyesters using different catalysts was calculated and presented in Figure 4a–e. As it was anticipated, the hydroxyl contents were reduced with increasing time and temperature during SSP, therefore, at higher SSP temperature (205 °C), the slowdown was the most important. The diagrams show a rapid decrease in the calculated –OH content during the first 2 h for all PEF polyesters regardless the type of catalyst. Afterwards, it becomes less important. This variation is in well agreement with the corresponding IV values, thus the latter shows an important increase during the first 2 h which leads to an important increase on the *M*_n_. At all times and temperatures investigated during SSP, the final PEF/Ge Ox polyesters exhibit the highest hydroxyl end-groups values (171.33 eq/10^6^) while PEF/Zr Is Ip displayed the lowest one (111.11 eq/10^6^). This is most likely due to the difference in the reactivity of the catalysts. Finally, using Sb Ac as a catalyst, PEF polyesters produced have the highest rate of inactive hydroxyl end-group, so it can be stated that this catalyst is the most active compared to the other four catalysts.

**Figure 5:**
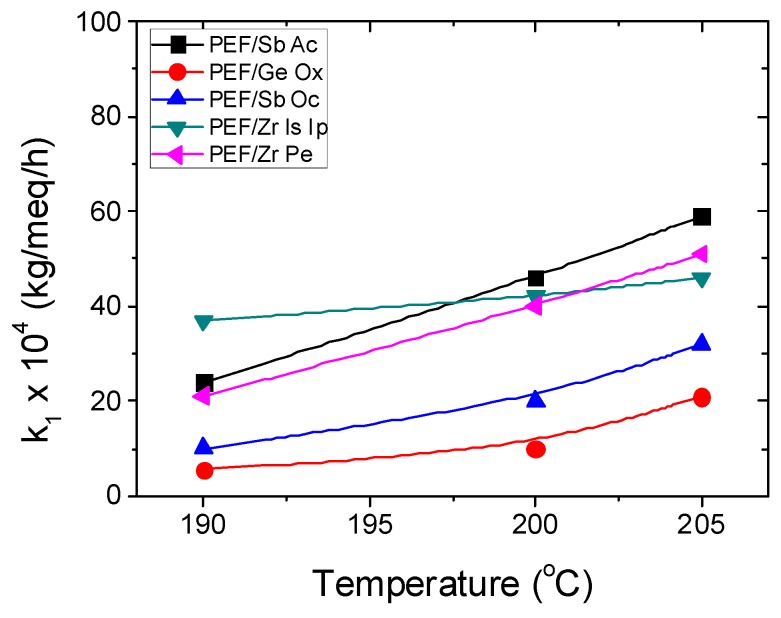

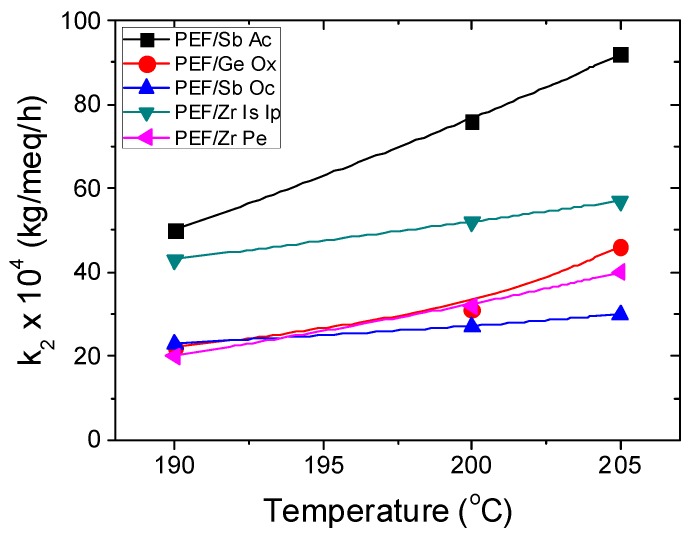
Variation of the estimated kinetic rate constants for polycondensation (*k*_1_) and esterification (*k*_2_) with temperature for all catalytic systems investigated.

Furthermore, the theoretical kinetic model presented in Section 3 was employed in order to estimate the kinetic rate constants of the esterification and polycondensation reactions, *k*_2_ and *k*_1_, respectively. Differential equations (7) and (8) were solved numerically together with equations (2), (9)–(11), and IV values, as well as the concentration of hydroxyl and carboxyl end-groups that were obtained as a function of SSP time. The parameters *k*_1_, *k*_2_, [OH]_I_, and [COOH]_i_ were calculated from fitting to the experimental data presented in Figure 1, Figure 2 and Figure 3 for all PEF samples at all temperatures and times. Optimized values are illustrated in Table 2 and plotted as a function of temperature in Figure 5. Results of the theoretical simulation curves are included as continuous lines in the above-mentioned figures. As it can be seen, theoretical simulation curves satisfactorily follow the experimental data at all different temperatures. Discrepancies could be attributed to the assumptions made, when developing the simple kinetic model.

From the data illustrated in Table 2 it seems that the values of the transesterification rate constant, k_1_, is lower than that of the esterification rate constant, k_2_, for all polyesters except PEF/Zr Pe, where similar values were estimated. The lower polycondensation rate constants, such as k_1_, were estimated for PEF/Ge Ox followed by PEF/Sb Ox. These catalysts also together with PEF/Zr Pe exhibited the lower esterification rate constants, *k*_2_. This is an indication that the effectiveness of these catalysts was lower compared to the other two investigated, i.e., PEF/Zr Is Ip and PEF/Sb Ac. It should be pointed here that the latter catalyst system (i.e., PEF/Sb Ac) exhibited always the higher k_2_ values followed by PEF/Zr Is Ip, which, even at 200 and 205 ℃, had the highest k_1_ values. This can be explained since the relative increase in the average molecular weight achieved by the Sb Ac catalyst, for example after 5h SSP at 205 ℃, was almost 200% compared to the corresponding of Zr Is Ip, which was almost 100%. Hence, it can be postulated that the Sb Ac catalyst provides the higher kinetic rate constants compared to all other catalysts, followed by Zr Is Ip.

In addition, from Table 2 it was estimated that the best fit values for the hydroxyl inactive groups, [OH]_i_, meaning those which are inaccessible to react, are always lower in PEF/Zr Is Ip compared to the other polyesters, while they always reduce with increasing temperature. An increase in temperature improves the mobility of the polymer chains and thus reduces the number of inactive end species. The lower values in PEF/Zr Is Ip are a direct consequence of the always lower –OH end-group concentration measured at all reaction times and temperatures compared to other polyesters (see Figure 4). Concerning the inactive [COOH]_i_, the values are always low enough and similar for all catalytic systems investigated.

Finally, both kinetic rate constants were correlated with temperature using an Arrhenius-type expression. As it is expected, the values of all rate constants increase with SSP temperature in accordance with the mobility and activity of the chain ends. When plotting ln(*k*) vs 1/*T*, good straight lines were obtained with a correlation coefficient greater than 0.90 (Table 3). From the slope of these straight lines the activation energies for the transesterification, *E*_1_, and esterification, *E*_2_, reactions were determined and illustrated in Table 3. Moreover, form the intercept of these straight lines the pre-exponential factors can be calculated and are also included in the same Table. It should be noted that the estimation of the activation energies using only three experimental data points (at the three investigated temperatures) results in a somehow great uncertainty in the values denoted by their high standard deviation. Thus, a safe conclusion concerning the activation energies cannot be set. However, there a definite indication that the PEF/Zr Is Ip system has a much lower *E*_1_ compared to all other systems and, an *E*_2_, which, within experimental error, seems to be also the lower one. In contrast the highest activation energy was measured when Ge Ox was used as a catalyst. This model results verify the fact that certainly Zr Is Ip exhibits the best catalytic characteristics compared to the other catalysts employed lowering the activation energy of both reactions involved during SSP.

### 4.2. Thermal Analysis of PEF Polyester Samples Prepared by Solid-State Polymerization

**Figure 6:**
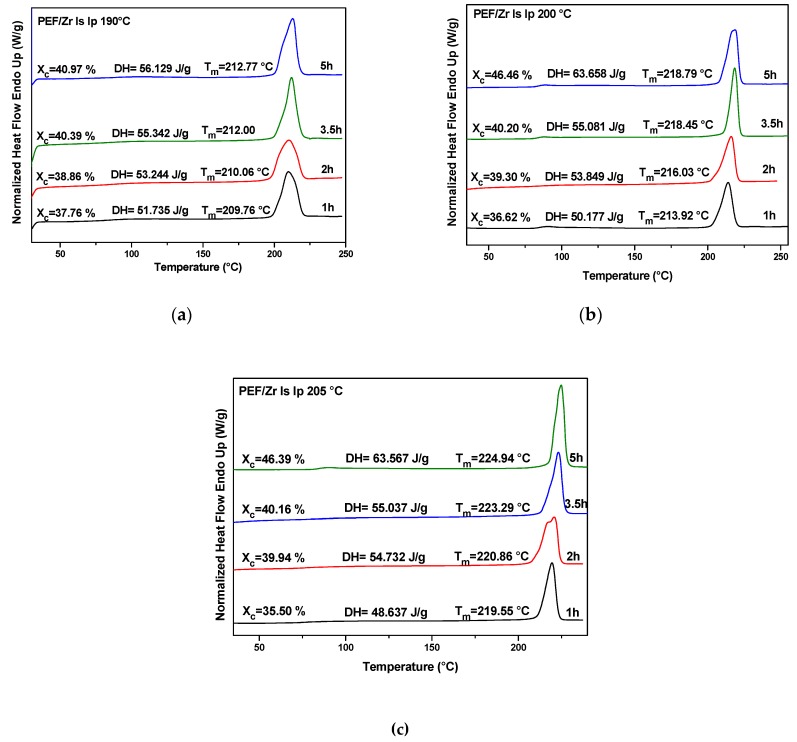
Differential scanning calorimetry (DSC) thermograms of different PEF/Zr Is Ip samples prepared after SSP at different temperatures and times: (**a**) 190 °C, (**b**) 200 °C, and (**c**) 205 °C.

The DSC results showed that the thermal properties of PEF were found to be obviously enhanced after the SSP procedure. Figure 6 and Figures S1–S4 in Supporting Information highlighted the melting behavior of PEF polyester samples at different reaction times and temperatures. As can be seen, regardless the catalyst type used, the endothermic melting peaks were shifted to higher values (increased *T*_m_) with increasing of either SSP time or SSP temperature, with an accompanying increase in the degree of crystallinity of resulting polymer. As expected, the increased molecular weight of the polyester sample during SSP process triggers the increase of the sharpness of the melting peaks as well as the increase in the melting points. Interestingly, DSC heating scans of PEF samples revealed double melting endotherms for PEF/Sb Ac, PEF/Ge Ox, and PEF/Zr Pe catalytic systems at the lowest SSP temperature (190°C) performed at *t*_ssp_ 1 h. This behavior is mainly time dependent and it has been completely vanished for PEF/Sb Ac and PEF/Ge Ox at 2h of SSP, while for PEF/Zr Pe, the double peaks have been merged to one after 3h. This outcome could be ascribed to crystals with different sizes and perfections induced by the thermal treatments of polymer as well as the annealing process [69,74,80,81,82]. This finding was recently reported in our previous work [70] for SSP process of PEF using three different catalysts, that is, titanium (IV) isopropoxide, dibutyltin (IV) oxide, and tetrabutyltitanate. Moreover, similar multiple melting peaks have been commonly detected during SSP in some alipharomatic polyesters like PET [85,86,87]. For PEF/Zr Is Ip, only one melting peak was recorded, which means that neither increasing SSP temperature nor time could change the behavior of the obtained polymer. Furthermore, PEF/Sb Ox exhibited double melting peaks after 2h SSP process and they did not disappear even after 5h. This is could be associated with the formation of two different types of persistent crystal formation. It is worth noting that the enthalpy of fusion (D*H*_m_) is also time- and temperature-dependent, that is, it increases progressively either increasing the SSP time or the SSP temperature.

**Figure 7:**
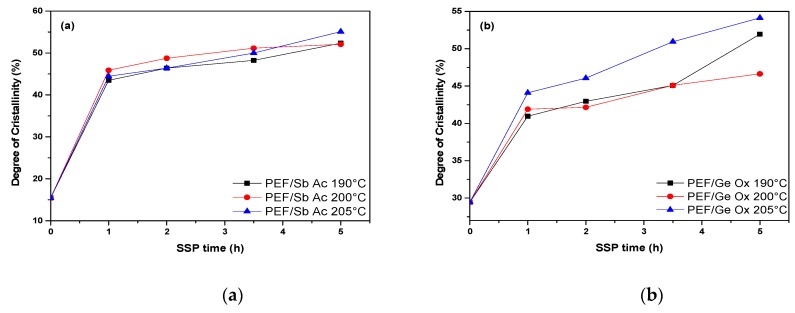

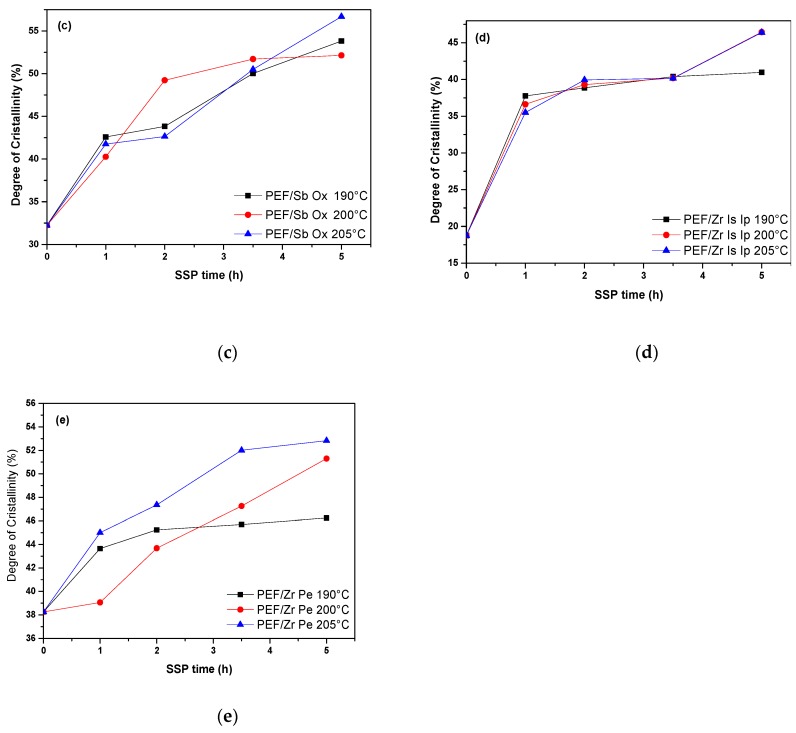
Effect of SSP time and temperature on the evolution of the degree of crystallinity of PEF samples: (**a**) PEF/Sb Ac, (**b**) PEF/Ge Ox, (**c**) PEF/Sb Ox, (**d**) PEF/Zr Is Ip, and (**e**) PEF/Zr Pe.

As the degree of crystallinity (*X*_c_) in semicrystalline polymers plays an important role in determining the final properties of resulting materials, that is, it has a direct bearing on the polymers optical, mechanical, chemical, and thermal properties, *X*_c_ for all prepared SSP PEF samples (presented in Table 4) was calculated from the measured melting enthalpy (D*H*_m_) values, using the heat of fusion value for the pure crystalline PEF that was reported in a previous study to be about 137 J·g^−1^ [55]. As depicted in Figure 7, whatever SSP time and temperature were used, the PEF/Zr Is Ip system exhibits much lower *X*_c_ values compared to those of other systems. PEF/Sb Ac, PEF/Ge Ox, PEF/Sb Ox, and PEF/Zr Pe have almost similar *X*_c_ values at 205°C, with a difference average up to 4%, while PEF/Zr Is Ip showed at the same temperature a much lower values, with respect to the latter four (difference average of 17%). It has been clearly illustrated in Table 4 that *X*_c_ follows the same trend of SSP temperature, that is, achieving the highest value at temperatures close to the melting point of PEF samples. This is associated with the higher macromolecular chain mobility and thereby higher folding ability to create crystalline lamellae. Since SSP occurs in the amorphous regions of the semi crystalline polyester, where end-groups are excluded from crystalline regions, it can thus be concluded that the rapidly increasing IV/molecular weight rate of PEF/Zr Is Ip, compared with the others four polyester samples, is due to its lowest degree of crystallinity, as indicated in Figure 7. The latter induces an increase in the chain mobility, which becomes less restricted, hence leading to a more facile diffusion rate of reaction by-products (ethylene glycol and water) by imposing a lower degree of resistance to mass transfer. Thus, this implies that molecular weight increase became faster. The *M*_n_/IV values obtained in the current study are in good accordance with this explanation.

## 5. Conclusions

Solid-state polymerization of the ecofriendly polyester PEF was investigated with a variety of catalysts at several temperatures. The effect of catalyst type, which is an important parameter influencing the SSP kinetics on molecular weight increase, of the resultant polyesters was discussed in detail, both experimentally and using a simple kinetic model. As SSP temperature and time increased, the intrinsic viscosity and the average molecular weight of PEF also increased. This is owing to the elimination of formed by-products during both the transesterification and esterification reactions that occurring during SSP, are diffusion-controlled. A simple kinetic model was also developed and used to get a deeper insight into the PEF polyester’s intrinsic viscosity as well as hydroxyl and carboxyl end-groups content by prediction their time evolution during SSP. From both the theoretical simulation results and the experimental measurements, it is proven that Zr Is Ip catalyst resulted in lower activation energies of both responsible reactions on the molecular weight increase during SSP. This finding explains the obtaining of higher molecular weight PEF samples with respect to those prepared using other catalysts. In contrast, the presence of the Ge Ox catalyst resulted in the highest activation energies, thus, leading to lower molecular weight PEF, as confirmed by IV measurement obtained herein. As an overall conclusion for the five catalysts used in the current work, Zr Is Ip displayed the best catalytic characteristics during SSP procedure.

## Figures and Tables

**Table 1:** Number average molecular weights (${\bar{M}}_{n}$, g/mol) of PEF polyester using different catalysts obtained after SSP at different temperatures and times. The value included in parenthesis is the corresponding number average degree of polymerization.

Temperature (°C)	SSP Time (h)	PEF/Sb Ac	PEF/Ge Ox	PEF/Sb Ox	PEF/Zr Is Ip	PEF/Zr Pe
	0	4000 (21)	4600 (25)	5700 (31)	8000 (44)	5000 (27)
190	1	7100 (39)	4600 (25)	6000 (33)	10300 (57)	7400 (41)
	2	7400 (41)	5700 (31)	7400 (41)	11300 (62)	8300 (46)
	3.5	7700 (42)	6800 (37)	8000 (44)	12400 (68)	8600 (48)
	5	9300 (51)	8300 (46)	8300 (46)	13100 (81	9000 (49)
200	1	7700 (42)	5400 (30)	7700 (42)	10300 (57)	10300 (57)
	2	9000 (49)	6500 (36)	8000 (44)	12400 (68)	11300 (62)
	3.5	10300 (57)	8000 (44)	8600 (48)	13800 (76)	12000 (66)
	5	11000 (60)	9000 (49)	10000 (55)	14400 (79)	12400 (68)
205	1	9000 (49)	6800 (37)	9300 (51)	12000 (66)	11000 (60)
	2	10000 (55)	9600 (53)	9600 (53)	13500 (74)	12000 (66)
	3.5	10600 (58)	10600 (58)	10300 (57)	15000 (82)	12400 (68)
	5	12000 (66)	11000 (60)	11700 (64)	15800 (86)	12700 (70)

**Table 2:** Kinetic rate constants of the transesterification and esterification reaction and concentration of temporarily inactivated OH and COOH end-groups at different polycondensation temperatures of PEF/Sb Ac, PEF/Ge Ox, PEF/Sb Ox, PEF/Zr Is Ip, and PEF/Zr Pe.

Sample	Temp. (°C)	k_1_ (kg/meq) h^−1^	k_2_ (kg/meq) h^−1^	[OH]_i_ (meq/kg)	[COOH]_i_ (meq/kg)
PEF/Sb Ac	190	24 × 10^−4^	50 × 10^−4^	162	13
	200	46 × 10^−4^	76 × 10^−4^	150	7.5
	205	59 × 10^−4^	92 × 10^−4^	138	6.0
PEF/Ge Ox	190	5.5 × 10^−4^	22 × 10^−4^	162	13
	200	10 × 10^−4^	31 × 10^−4^	150	10
	205	21 × 10^−4^	46 × 10^−4^	138	6.0
PEF/Sb Ox	190	10 × 10^−4^	23 × 10^−4^	162	13
	200	20 × 10^−4^	27 × 10^−4^	150	9.5
	205	32 × 10^−4^	30 × 10^−4^	138	6.0
PEF/Zr Is Ip	190	37 × 10^−4^	43 × 10^−4^	105	13
	200	42 × 10^−4^	52 × 10^−4^	96	10
	205	46 × 10^−4^	57 × 10^−4^	91	6
PEF/Zr Pe	190	21 × 10^−4^	20 × 10^−4^	162	13
	200	40 × 10^−4^	32 × 10^−4^	136	10
	205	51 × 10^−4^	40 × 10^−4^	130	4.5

**Table 3:** Activation energies, pre-exponential factors, and correlation coefficients of the transesterification and esterification reaction of all PEF polyesters.

Sample	*E*_1_ (kJ/mol)	ln(*k*_01_ × 10^4^)	*R* ^2^	*E*_2_ (kJ/mol)	ln(*k*_02_ × 10^4^)	*R* ^2^
PEF/Sb Ac	111.6 ± 6.1	32.18 ± 1.55	0.994	75.1 ± 1.1	23.41 ± 0.27	0.999
PEF/Ge Ox	158.7 ± 19.6	42.88 7.28	0.937	86.3 ± 15.1	25.48 5.35	0.898
PEF/Sb Ox	140.3 ± 12.3	38.72 ± 3.14	0.985	32.3 ± 2.5	11.47 ± 0.65	0.987
PEF/Zr Ip Is	26.2 ± 2.7	10.40 ± 0.70	0.979	34.6 ± 0.1	12.75 ± 0.01	0.999
PEF/Zr Pe	110.2 ± 6.3	31.67 ± 1.62	0.993	85.2 ± 0.4	25.11 ± 0.11	0.999

**Table 4:** Degree of crystallinity values (%) of the SSP PEF samples using different catalysts.

SSP Temperature (°C)	SSP time (h)	PEF/Sb Ac	PEF/Ge Ox	PEF/Sb Ox	PEF/Zr Is Ip	PEF/Zr Pe
	0	15.43	29.44	32.23	18.76	38.25
190	1	43.49	40.96	42.59	37.76	43.65
	2	46.38	42.96	43.83	38.86	45.24
	3.5	48.25	45.08	50.03	40.39	45.70
	5	52.35	51.95	53.83	40.97	46.24
200	1	45.88	41.90	40.27	36.62	39.06
	2	48.74	42.15	49.24	39.30	43.68
	3.5	51.15	45.09	51.72	40.20	47.26
	5	52.07	46.64	52.15	46.46	51.30
205	1	44.46	44.10	41.76	35.50	45.01
	2	46.39	46.07	42.65	39.94	47.36
	3.5	50.00	50.94	50.50	40.16	52.01
	5	55.10	54.13	56.70	46.39	52.83

## Supplementary Materials

The following are available online at https://www.mdpi.com/2073-4360/11/3/438/s1, Figure S1: DSC thermograms of PEF/Sb Ac samples prepared after SSP at different temperatures and times, Figure S2: DSC thermograms of PEF/Ge Ox samples prepared after SSP at different temperatures and times. Figure S3: DSC thermograms of PEF/Zr Pe samples prepared after SSP at different temperatures and times. Figure S4: DSC thermograms of PEF/Sb Ox samples prepared after SSP at different temperatures and times.
