# Supplementary material for: Solid-State Polymerization of Poly(Ethylene Furanoate) Biobased Polyester, III: Extended Study on Effect of Catalyst Type on Molecular Weight Increase
Source: Polymers (Basel). 2019 Mar 6;11(3):438. doi: 10.3390/polym11030438 (PMC6473661; doi:10.3390/polym11030438)
Supplement: Supplementary file 1 [file polymers-11-00438-s001.pdf]

polymers

MDPI

Supplementary

Solid-State Polymerization of Poly(Ethylene Furanoate) Biobased Polyester, III: Extended Study on Effect of Catalyst Type on Molecular Weight Increase

Yosra Chebbi [1,2], Nejib Kasmi [2], Mustapha Majdoub [1], George Z. Papageorgiou [3,*], Dimitris S. Achilias [2] and Dimitrios N. Bikiaris [2,*]

[1] Laboratoire des Interfaces et Matériaux Avancés, Université de Monastir, 5000 Monastir, Tunisia; yossrachebbi@gmail.com (Y.C.); mustaphamajdoub@gmail.com (M.M.)
[2] Laboratory of Polymer Chemistry and Technology, Department of Chemistry, Aristotle University of Thessaloniki, GR-541 24, Thessaloniki, Macedonia, Greece; nejibkasmi@gmail.com (N.K.); axilias@chem.auth.gr (D.S.A.)
[3] Chemistry Department, University of Ioannina, P.O. Box 1186, 45110 Ioannina, Greece
* Correspondence : dbic@chem.auth.gr (D.N.B.); gzpap@cc.uoi.gr (G.Z.P.); Tel.: +30-231-0997812 (D.N.B.); +30-265-1008354 (G.Z.P.)

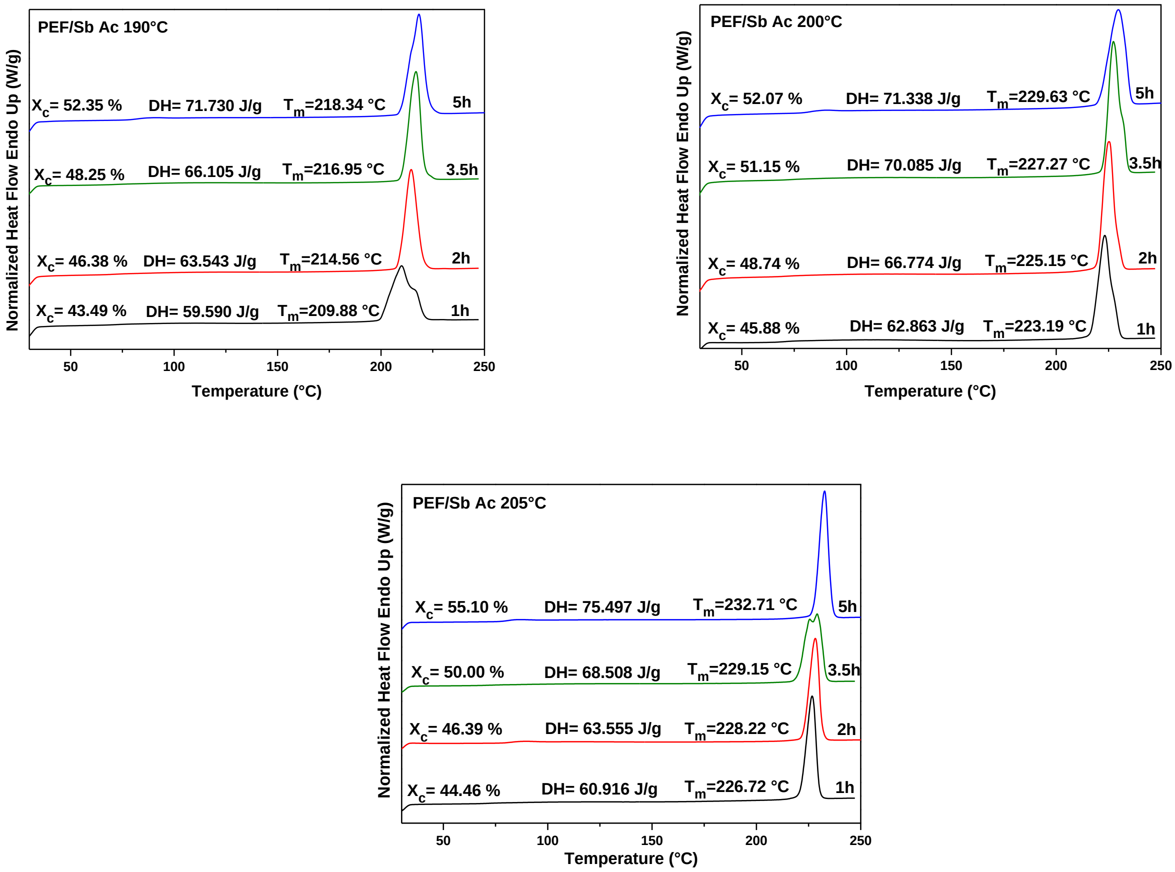

Figure S1. DSC thermograms of PEF/Sb Ac samples prepared after SSP at different temperatures and times.

polymers

MDPI

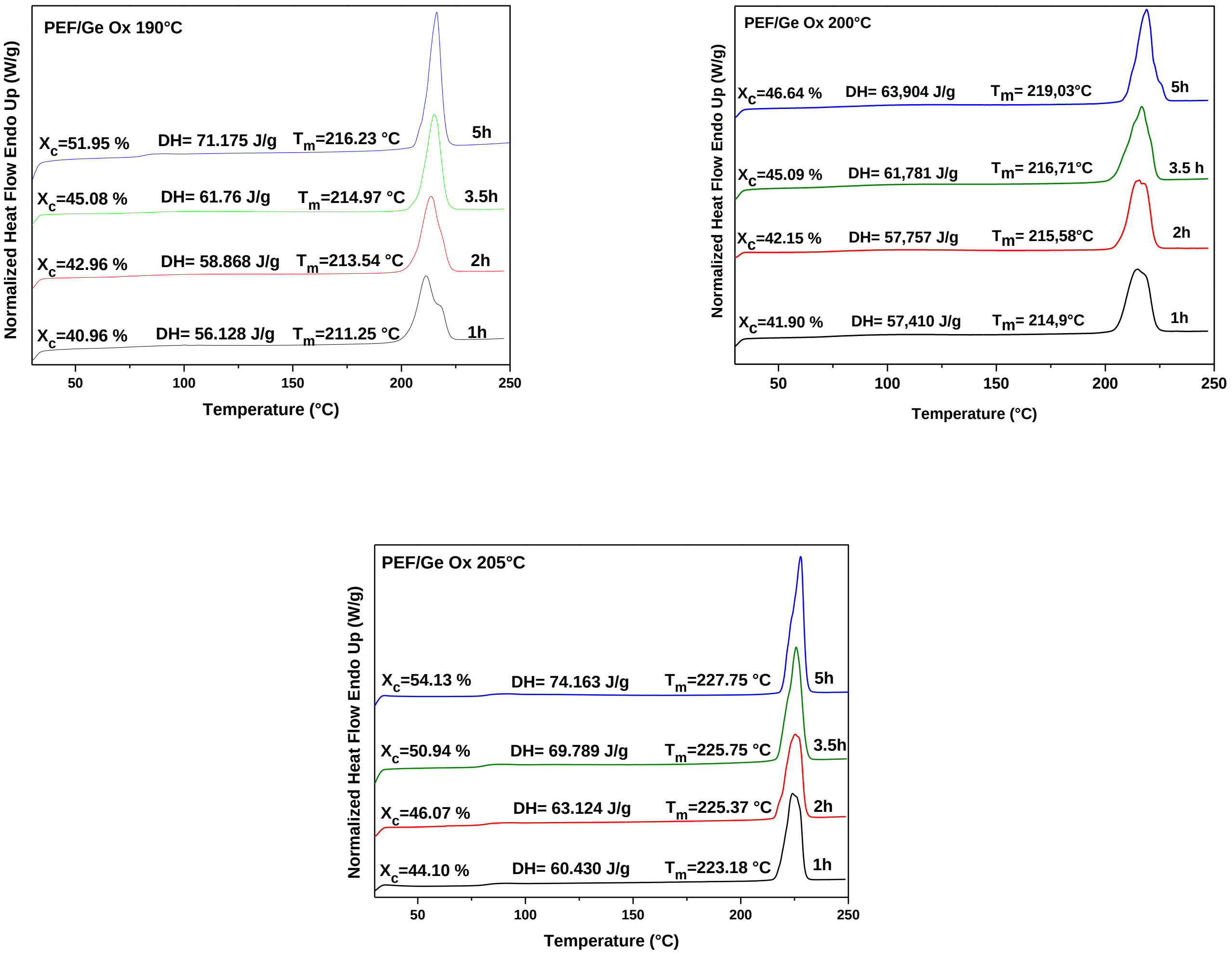

Figure S2. DSC thermograms of PEF/Ge Ox samples prepared after SSP at different temperatures and times.

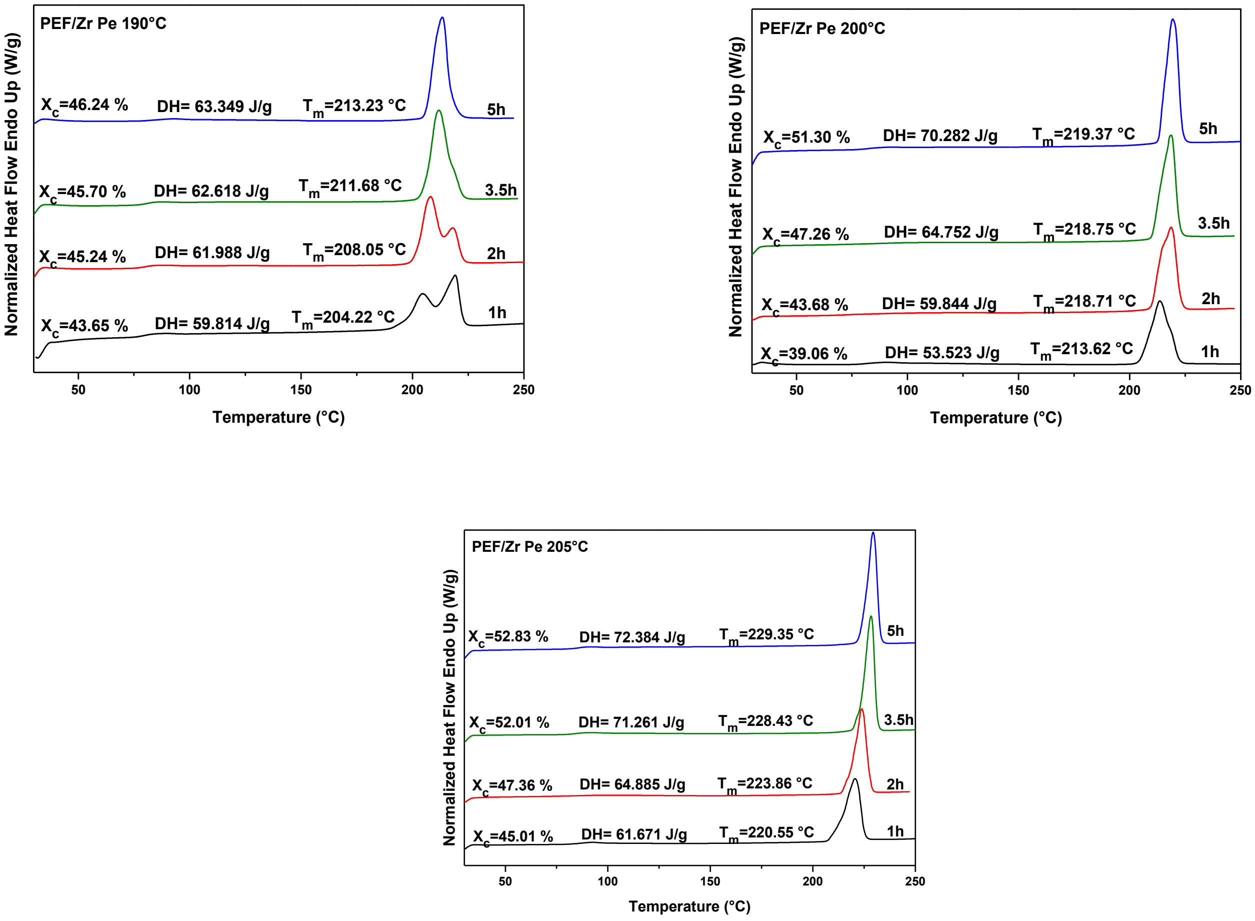

Figure S3. DSC thermograms of PEF/Zr Pe samples prepared after SSP at different temperatures and times.

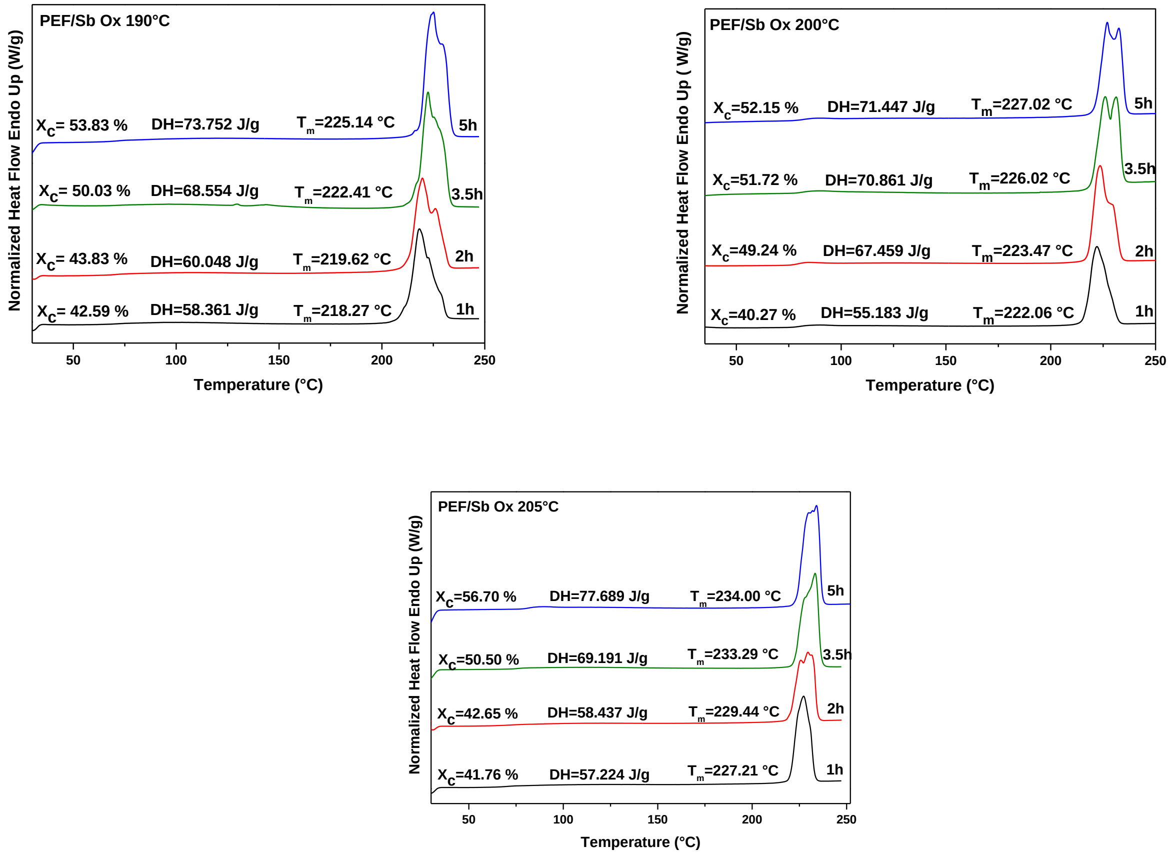

Figure S4. DSC thermograms of PEF/Sb Ox samples prepared after SSP at different temperatures and times.
